# The Effects of 6 Isocaloric Meals on Body Weight, Lipid Profiles, Leptin, and Adiponectin in Overweight Subjects (BMI > 25)

**Published:** 2014-04-01

**Authors:** Zeynab Hatami Zargaran, Moosa Salehi, Seyed Taghi Heydari, Siavash Babajafari

**Affiliations:** 1 Research Center of Nutrition and Food Sciences, Shiraz University of Medical Sciences, Shiraz, IR Iran; 2 Research Center for Cardiovascular Atherosclerosis, Jahrom University of Medical Sciences, Jahrom, IR Iran

**Keywords:** Obesity, Meal, Leptin, Adiponectin

## Abstract

**Background:**

It seems that meal frequency is negatively related to body weight, but the relationship between meal frequency and weight loss is not clearly known yet.

**Objectives:**

The present study aimed to investigate whether 6 isocaloric meals affected body weight, lipid profiles, leptin, and adiponectin in overweight subjects.

**Methods:**

The present randomized controlled trial was conducted on 90 overweight subjects in 3 months. The subjects were randomly divided into two groups. The control group continued their normal diet, while the intervention group was required to follow a 6 isocaloric meal diet instead of their previous meal pattern (3 meals and 2 snacks). The planned reduced calorie diets for both groups were identical, except for meal pattern. Blood samples were analyzed prior to and at the end of the study for total cholesterol, triglyceride, HDL-C, LDL-C, leptin, and adiponectinn concentrations. Paired t-test was used for comparison of the measurements before and after the study in each group. Besides, independent t-test was used for comparison of the measurements between the groups. P value less than 0.05 was considered as statistically significant.

**Results:**

The mean age of the participants was 36.38 ± 9.7 in the intervention group and 37.6 ± 10.9 in the control group. In comparison to the control group, the intervention group showed a significant decrease in total cholesterol (P < 0.001), LDL-C (P < 0.001), BMI (P < 0.001), triglyceride (P < 0.001), and leptin (P = 0.002) and a significant increase in HDL (P < 0.001) and adiponectin (P = 0.031).

**Conclusions:**

The 6 isocaloric meal pattern led to a reduction in BMI, lipid profiles (total cholesterol, LDL-C, triglyceride), and leptin concentrations and an increase in HDL and adiponectin compared to the normal diet.

## 1. Background

Although there is a strong interest in losing weight, the prevalence of overweight and obesity is rising around the world. Energy balance equation shows that weight gain can occur only when energy intake is greater than energy expenditure for a prolonged period (1). In the recent years, the significant increase in the prevalence of overweight and obesity in both developed and developing countries shows that the origin of this public health problem is complex and multifactorial (2-7).

The prolonged persistence of energy imbalance between energy intake and energy expenditure, such as a sedentary lifestyle, macronutrient composition of the diet, and increased energy density of foods, has induced weight gain. Of course, environmental and behavioral changes, such as changes in dietary habits, may also be accountable for the growth of obesity in the world (8).

Obesity is actually a precursor to many diseases, such as diabetes mellitus, cancer, coronary heart disease, and sleep-breathing disorders (9). One of the proposed methods for weight loss and appetite control is increasing the frequency of meals, regarding which there is controversy among many researchers (3, 10, 11).

The number of meals in different countries is affected by cultural and social beliefs as well as an individual’s personal beliefs about health and body composition (12).

Leptin and adiponectin are important enzymes that are associated with obesity. Leptin is an enzyme that is secreted from white adipose tissue and its concentration depends on the degree of obesity, being higher in obese individuals (13). Additionally, adiponectin is an adipocyte-specific protein the serum levels of which are lower in obese individuals. Frequent meals can also reduce the lipid profiles, leptin, and adiponectin, but its effect are yet unproven (14-16).

## 2. Objectives

The present study aims to assess the effects of daily energy intake in 6 isocaloric meals in comparison to the current traditional diet (with 3 meals and 2 small snacks per day) on weight, lipid profiles, leptin, and adiponectin for 3 months.

## 3. Patients and Methods

Sampling began after receiving permission from the Ethics Committee of Shiraz University of Medical Sciences. At first, 120 subjects volunteered to participate in this study and 90 ones were selected based on the inclusion criteria. The inclusion criteria of the study were, being between 20 and 60 years old, not taking cholesterol-lowering drugs, not being on lipid-lowering or weight control diets, being free from possible causes of secondary hypercholesterolemia (e.g., hyperthyroidism, pregnancy), not working night shifts, not smoking, and not suffering from chronic diseases (e.g., cancer, renal failure, heart failure). Written informed consents to participate in the study were obtained from all the participants. It should be mentioned that the subjects did not pay any fees to participate in the study. In this study, four 24-hour dietary recalls, including assessments of food intake at the beginning and end of the study and at the end of each month, were obtained from all the participants.

### 3.1. Study Design and Diets

By 6 iso-caloric meals, we mean that energy is divided into six equal parts (one meal every three hours). For example, if the total energy of one day is 1800 kcal, 300 kcal per serving should be taken with equal intervals.

In the present randomized controlled trial, the participants were randomly divided into an intervention and a control group. At the beginning, the weight loss diet was written for both groups. The calorie intake of the planned diets for each subject was 400 kcal less than the amount of energy needed for weight maintenance and the percentage of energy from macronutrients was the same in both groups. The control group continued their normal diet (most of their diet consisted of three meals and two snacks), while the intervention group had to follow a 6 isocaloric meal diet instead of their previous meal pattern. The intervention group was trained on how to divide daily energy into 6 isocaloric meals in 4 sessions. On the other hand, the control group was trained only about healthy nutrition in 2 sessions.

The participants’ height and weight were measured by standard anthropometric methods at the beginning, in the middle, and 3 months after the study. Weight was measured to the nearest 0.1 kg on a Seca electronic scale when the subjects were fasting, were wearing light clothes, and were not wearing shoes. In addition, height was measured to the nearest 0.1 cm by using a stadiometer. During the study, 2 subjects in the intervention group and 1 in the control group were excluded from the study because of their personal problems. Also, three subjects (1 in the control group and 2 in the intervention group) were excluded because of not following the planned diet. All the excluded participants were female.

### 3.2. Biological Sample Collection and Analysis

Venous blood samples were taken using venous retention needles prior to and at the end of the study. The samples were then analyzed for total cholesterol, triglyceride, HDL-C, LDL-C, leptin, and adiponectin concentrations. Moreover, lipid profiles (plasma total cholesterol, HDL-C, LDL-C, and triglycerides) were measured based on the photometric method (Autoanalyser BT 1500). Besides, serum adiponectin concentrations were measured using ELISA assay (Boster Human Adiponectin ELISA kit). Also, serum leptin concentrations were measured using a competitive ELISA kit (IBL GESELLSCHAFT FÜR IMMUNCHEMIE UND IMMUNBIOLOGIE MBH, Germany).

To eliminate day-to-day laboratory variations, all the blood samples were analyzed in a single batch following the completion of the study.

### 3.3. Statistical Analysis

The data were analyzed using the SPSS statistical software (version 16). Paired t-test was used for comparison of the measurements before and after the study in each group. Additionally, independent t-test was used for comparison of the measurements between the groups. P value less than 0.05 was considered as statistically significant. In order to avoid obtaining negative values, for all the variables except for adiponectin and HDL, the values obtained at the beginning of the intervention were subtracted from those obtained at the end.

## 4. Results

According to the study results, 18 participants (80%) were female, 61% had academic degrees, 55% were currently employed, and 70% were currently married. Moreover, 8 out of the 45 participants in the intervention group and 10 out of the 45 participants in the control group were male and the rest were female. The mean BMI was 30.9 ± 5.1 in the intervention group and 30.3 ± 4.7 in the control group (P > 0.05). The results revealed significant changes in weight, lipid profiles, leptin, and adiponectin in the intervention group (P < 0.05); such a way that HDL and adiponectin were increased, while weight, total cholesterol, LDL, TG, and leptin were decreased. In the control group, on the other hand, increase in HDL and decrease in leptin were not statistically significant (P > 0.05). However, a significant reduction in other lipid profiles and a significant increase in adiponectin were observed in this group (P < 0.05). In comparison to the control group, a significant increase in adiponectin and HDL and a significant decrease in BMI and lipid profiles were observed in the intervention group (P < 0.05) (Table1). Furthermore, a significant change was found in all the risk factors in the intervention group compared to the control group (P < 0.05) (Table 2). For instance, LDL was reduced by 24.4 ± 14.5 mg / dL in the intervention group and by 7.7 ± 15.3 mg / dL in the control group (P < 0.001).

**Table 1.** Changes in the Study Variables in Response to Meal Pattern

Variable		Intervention Group			Control Group		
		Mean	SD	P value	Mean	SD	P value
**LDL (mg / dL)**	Before	134.1	34.6	< 0.001	126.5	30.9	0.002
	After	109.8	31.0		118.8	28.5	
**HDL (mg / dL)**	Before	38.6	5.4	< 0.001	40.8	5.5	0.063
	After	41.2	5.6		41.4	5.7	
**CHOL (mg / dL)**	Before	183.9	34.7	< 0.001	191.4	40.3	0.020
	After	160.7	31.4		184.3	37.9	
**TG (mg / dL)**	Before	156.5	68.0	< 0.001	162.2	59.7	0.007
	After	134.4	61.5		157.2	57.6	
**BMI (kg / m**^**2**^**)**	Before	30.9	5.2	< 0.001	30.3	4.7	< 0.001
	After	28.9	4.9		29.5	4.3	
**Adiponectin (μg / mL)**	Before	16.3	3.7	< 0.001	18.1	3.5	0.036
	After	18.9	3.8		19.1	3.7	
**Leptin (mg / mL)**	Before	7.6	1.2	< 0.001	6.9	1.6	0.129
	After	6.2	1.4		6.5	1.5	

**Table 2.** Compression of the Differences in the Two Groups in Response to Meal Pattern

	Intervention Group (n = 45)	Control Group (n = 45)	P value
**Difference in LDL**	24.4 (14.5)	7.7 (15.3)	< 0.001
**Difference in HDL**	2.5 (1.7)	0.5 (1.9)	< 0.001
**Difference in CHOL**	23.2 (14.9)	12.0 (19.9)	< 0.001
**Difference in TG**	22.1 (23.5)	4.9 (11.7)	< 0.001
**Difference in BMI**	1.9 (0.9)	0.7 (1.1)	< 0.001
**Difference in Adiponectin**	2.6 (3.2)	1.0 (3.3)	0.031
**Difference in Leptin**	1.4 (1.6)	0.3 (1.5)	0.002

## 5. Discussion

This study explored the relationship between meal frequency and BMI, lipid profiles, leptin, and adiponectin in an obese population. Due to the increased prevalence of overweight and obesity in the society and the risk of chronic diseases associated with obesity, it is important to identify ways to reduce weight. Perhaps one of the advisable factors for weight control is increasing the frequency of meals. The results of our study indicated a relationship between the 6 isocaleric meal pattern and improvement of lipid profiles and BMI.

There are four assumptions according to which an increased frequency of meals could help control weight. First, increased frequency of meals may help control hunger (Burley et al. (17) and Westerterp-Plantenga et al. (18)). Second, increased frequency of meals may increase dietary carbohydrate: fat ratio (Drummond et al. 1996 (19)). Third, increased meal frequency may shift some of the energy towards the earlier part of the day. Some studies showed that obese individuals skipped breakfast and much of their energy was received in the afternoon. (Bellisle et al. (20) and Fricker et al. (21)). Fourth, a dietary pattern of “little and often” may be more consistent with an active lifestyle compared to two or three servings of food pattern (Kirsch and von Ameln (22)).

About 50 years ago, a discussion began regarding the relationship between meal frequency and weight control (14). However, the large number of articles supporting and opposing the theory of an inverse relationship between increased frequency of meals and weight loss makes it difficult to confirm the theory.

In this study, both groups had significant weight loss and the weight loss was more significant in the intervention group. In agreement with these findings, many studies, including those conducted by Ma et al. (10) and Franko et al. (23), have supported the effectiveness of increased frequency of meals in weight loss. Moreover, Ruidavets et al. (24) performed a study on 330 males (45 - 94 years old) and revealed a significant negative correlation between eating frequency and BMI.

However, in the study Cameron et al. conducted on the effects of 6 isocaloric meals on obese men and women for 8 weeks, the weight loss in the two groups was not statistically significant (25). These results are similar to those obtained by Forslund et al. (26), Pearcey and de Castro (27), and Yannakoulia et al. (28) all of whom refuted the effectiveness of increased meal frequency on weight loss. The insignificant weight loss in the intervention group in these studies might be due to the fact that a larger number of meals was required the sample size was insufficient, or the intervention group did not consume exactly 6 isocaloric servings. Aside from genetic differences between the individuals participating in the study, there are other confounding factors that could change the results of the studies. The participants of the current study had approximately average levels of education and economic conditions, while the participants of other studies might have been recruited from different conditions.

Increased frequency of meals has been shown to have health promoting benefits on lipid profiles in obese populations. For instance, Juhel et al. (29) showed that the individuals with greater fat and cholesterol intakes might profit more from increased frequency of meals. The results of the present study also confirmed that the 6 isocaloric meal pattern led to improvement of lipid profiles. In this study, the increase in HDL and the decrease in other lipid profiles were more significant in the intervention group compared to the control group. These results are similar to those of many other studies. In a study performed by Edeistein et al. (30) on 2,034 men and women, those who reported eating more frequently had significantly lower cholesterol and LDL concentrations in comparison to those with an infrequent meal pattern.

Some evidence suggests that the cholesterol-lowering effects of meal frequency were not dependent on weight. The findings of a study which was conducted on 11 obese men (31) showed a higher cholesterol level in the group which consumed one serving per day in comparison to the other two groups (3 meals and 6 meals a day). In the same line, the results of another study (32) performed on 39 men showed that men had lower cholesterol concentrations when they consumed 8 meals a day instead of 3 meals, while their body weight was stable. These results indicate that it is the increased frequency of meals and not body weight that reduces blood cholesterol concentrations. The results of our study were similar to those of the study by Titan et al. (33) who showed an inverse relationship between the serum lipid profiles and increased frequency of meals.

An important finding is that increased frequency of meals will cause a change in the quality of life. Increasing the number of meals per day will lead to increased energy intake from carbohydrates, decrease in energy intake from fat and protein, increased fiber intake, higher consumption of fruit and vegetables, and increase in ascorbic acid and folate intake (10). Jenkins et al. (34) indicated that high frequency intake of food would help reduce cholesterol because a smaller carbohydrate meal reduces insulin secretion consequently reducing HmG-CoA reductase, an enzyme which is responsible for hepatic cholesterol synthesis. Moreover, decrease in cholesterol synthesis increases LDL receptors, thereby lowering LDL and total cholesterol concentrations (34). It is possible that those who consume fewer servings per day (perhaps because of skipping breakfast) have higher cholesterol concentrations (35). The Alameda County study reported that breakfast consumption was associated with a reduction in the mortality risk (35).

The findings of the present study revealed a significant difference between the two groups regarding the increase in adiponectin. Also, the reduction in leptin concentrations was more significant in the intervention group compared to the control group. These results were on the contrary to those obtained by Dixit et al. (36) showing that meal frequency had no significant effects on adiponectin concentrations. In a study conducted by Carlson et al. (14) also, no significant correlation was found between serum adiponectin and leptin concentrations and frequency of meals. Furthermore, Kassab et al. (37) indicated that an increase in serum leptin was significantly associated with increased serum insulin. Besides, Jenkin et al. (34) demonstrated that mean insulin levels were 28% lower after following a 17-snack meal for 2 weeks compared to the three meal diet.

Duration of the present study and the number of participants were also suitable to investigate the relationship between the frequency of meals and indicators of good health. However, our results cannot be generalized to other social, cultural, and economic groups. Hence, further studies are required to assess the effects of meal frequency on weight loss. The relationship between meal frequency and other measures of interest, such as serum insulin concentrations, should also be considered in future studies.

The 6 isocaloric meal pattern reduced BMI, lipid profiles (total cholesterol, LDL-C, triglyceride), and leptin concentrations and increased HDL and adiponectin compared to the normal diet.
